# Exploring Gender Differences in Student Evaluations of Faculty in a Graduate Clinical Translational Research Program

**DOI:** 10.15694/mep.2020.000043.1

**Published:** 2020-03-11

**Authors:** Alexandra J Greenberg-Worisek, Katherine E. Cornelius, Karen Weavers, Felicity Enders

**Affiliations:** 1 Mayo Clinic College of Medicine and Science

**Keywords:** research education, gender disparities, faculty, evaluations, graduate

## Abstract

This article was migrated. The article was marked as recommended.

There has long been an underrepresentation of women in medicine and biomedical research; this is often described as a “leaky pipeline,” as the more senior the level of rank, the fewer women are appointed. In evaluating a faculty member for promotion, student evaluations of faculty teaching are often considered; therefore, if gender biases are reinforced by student evaluations of teaching, the gender gap in faculty promotion could remain or increase. In this study, we examine student evaluations of faculty teaching in a graduate biomedical research training program, using data gathered during two academic years. While female faculty received higher quantitative ratings of teaching, subtle gender differences in language existed in the student comments, indicating that implicit biases about women may be present in student evaluations of faculty teaching.

## Introduction

There has long been an underrepresentation of women in medicine and biomedical research. This gender gap is often attributed to the so-called “leaky pipeline”; that is, the further along in a career in medicine or biomedical research one goes, the greater the attrition of women in the field ( Villablanca and Howell, 2016). These various “leaks” occur at multiple points in the career trajectory for those in biomedical research, beginning with lower rates of women admitted during the graduate education admission process; additional “leaks” are found during the processes of faculty selection, promotion, and retention ( Liu *et al.*, 2019). Most of these differences are perceived from the top-down (that is, leadership levels), as the gender gap widens with increasing levels of promotion. Reasons for women leaving biomedical research are highly varied; however, many issues often cited are related to implicit and explicit bias, hostile work environments, sexual harassment, and other climate issues ( Kacik, 2019).

To date, little has been done to explore whether there are differences in how female faculty in graduate biomedical training programs are perceived by their students. As student evaluations of faculty are often considered when determining whether to promote a faculty member, understanding whether the gender gap is perceived and/or reinforced by students’ own gender stereotypes and implicit biases ( Burns-Glover and Veith, 1995). It has been established at the undergraduate level that student evaluations of faculty teaching are often rife with gender bias that is observable both in quantitative ratings of faculty and in open-text comments (gendered language) ( Sprague and Massoni, 2005; Basow *et al.*, 2006; MacNell *et al.*, 2015). Research has shown that differences exist in student evaluations of faculty in medical schools; specifically, female faculty receive lower teaching evaluations ( Morgan *et al.*, 2016); however, this phenomenon has never been explored in graduate biomedical research education.

In this study, we examined student evaluations of faculty teaching in a translational biomedical research program, using historical data gathered as standard of practice over the past two years. Based on prior work in related medical and health professions education, we hypothesized that women would receive lower teaching evaluation grades than their male counterparts, and that this would be even further pronounced between faculty with PhDs versus MDs. Additionally, we examined whether the composition of teaching teams (male-male, female-male, female-female) had any effect on student evaluation grades.

## Methods

### Program Description

For this study, faculty evaluations were used from all courses offered through Mayo Clinic Center for Clinical and Translational Science (CCaTS) track within the Mayo Clinic Graduate School of Biomedical Sciences (MCGSBS) for the 2017-2018 and 2018-2019 academic years. The program includes approximately ~52 unique courses delivered once or twice a year, or every other year, with each course having between 1 and 3 course directors. Coursework includes content across the clinical translational spectrum, from basic laboratory and clinical research to population and policy-focused work.

### Student Evaluation Data

This study utilized data gathered as part of the standard CCaTS post-course evaluation process. A standard evaluation form is sent to students at the end of each quarter for each course in which they are enrolled. The evaluation is anonymous and optional, though with high completion rates. The primary item of interest for this study was “Please assign a grade for the co-director, [name here], and comment on what you liked or can be improved.” This item included both quantitative data (assigning faculty a grade) and qualitative data (commenting on areas for improvement in an open text box). Students could assign faculty a letter grade (A, B, C, D, F), which was assigned a corresponding number on a Likert scale (4, 3, 2, 1, and 0, respectively). Data were used in aggregate (average for each course delivery) for each faculty member; that is, each faculty member received a mean grade for each course, yielding faculty*course units.

### Faculty Information

For each of the courses included, faculty degree, rank, gender, and team composition were gathered and incorporated into student evaluation feedback. Several faculty members teach multiple courses; this was taken into account during data cleaning and analysis. Once data were merged for the quantitative and qualitative data sets, faculty names and course numbers were removed.

### Data Analysis

Quantitative data were analyzed to generate descriptive statistics, and to test for differences between males and females using chi-squared/fisher’s exact and/or one-way and two-way ANOVA tests, depending on the variable and data type.

For qualitative data, two reviewers separately coded each as having gendered language (Y/N); containing positive, negative, or mixed feedback; and key words (adjectives, adverbs specifically) were noted.

## Results/Analysis

There were 57 unique faculty members who taught at least one course during the 2017-2018 and 2018-2019 academic years; a total of 88 course offerings were delivered within the CCaTS program during those two years ( Table 1). As most courses are team-taught by 2 or 3 faculty members, this yielded 144 faculty*course units. There were statistically significant differences in terms of highest degree attained by gender (p=0.02). Of these, 24 (42%) were female and 33 (58%) were male. Among the 32 faculty with a PhD, 19 (59.4%) were female and 13 (40.6%) were male; among the 17 faculty with MDs, 4 (23.5%) were female and 13 (76.5%) were male. There were no female faculty with dual MD/PhD degrees; however, there were 3 male faculty members who held both.

There were also statistically significant differences in rank by gender (p=0.018; Table 1). Of the 10 who held the rank of Assistant Professor, 8 (80%) were female; 7 of the 14 (50%) who held the rank of Associate Professor were female; and only 6 of 24 (25%) of individuals who held the rank of full Professor were women. There were not enough data to analyze fields of rank for each faculty member; however, more women held rank in epidemiology and health services researchers than men, and more men held rank in medicine. Notably, biostatistics was fairly evenly split (5 [45.5%) female and 6 [54.5%] male).

**Table 1.** Demographic characteristics of unique faculty within the Clinical Translational Science Track at Mayo Clinic Graduate School of Biomedical Sciences who taught a course in the 2017-2018 and/or 2018-2019 academic years (n=57).

	Female n (%)	Male n (%)	p-value
**Total**	24	33	
**Highest Degree**			0.020 *
*Masters*	1 (20.0%)	4 (80.0%)	
*MD*	4 (23.5%)	13 (76.5%)	
*PhD*	19 (59.4%)	13 (40.6%)	
*MD/PhD*	0 (0%)	3 (100.0%)	
**Rank**			0.018 *
*Instructor*	0 (0%)	3 (100%)	
*Assistant Professor*	8 (80.0%)	2 (20.0%)	
*Associate Professor*	7 (50.0%)	7 (50.0%)	
*Professor*	6 (25.0%)	18 (75.0%)	
*NA*	3 (50.0%)	3 (50.0%)	
**Primary Field of Rank**			0.149 *
*Anesthesiology*	0 (0%)	2 (100%)	
*Biochemistry and Molecular Biology*	0 (0%)	2 (100%)	
*Biomedical Ethics*	0 (0%)	3 (100%)	
*Biomedical Informatics*	0 (0%)	1 (100%)	
*Biostatistics*	5 (45.5%)	6 (54.5%)	
*Epidemiology*	5 (83.3%)	1 (16.7%)	
*Health Services Research*	4 (66.7%)	2 (33.3%)	
*Medical Education*	1 (100%)	0 (0%)	
*Medicine*	3 (27.3%)	8 (72.7%)	
*Neurology*	0 (0%)	2 (100%)	
*Pediatrics*	1 (50.0%)	1 (50.0%)	
*Pharmacology*	1 (50.0%)	1 (50.0%)	
*Physiology*	0 (0%)	1 (100%)	
*Psychology*	2 (100%)	0 (0%)	
*NA*	2 (40.0%)	3 (60.0%)	

* Fisher’s Exact Test used (Pr <= P)

Overall, there was a borderline statistically significant difference (p=0.051) in faculty grades between female and male faculty members, with female faculty members scoring slightly higher ( Table 2). No statistically significant difference was found when adding highest degree earned to the analysis (p = 0.647; Table 2). However, when examining the composition of the teaching team for each class, significant differences were seen in grades, with male course directors teaching solo earning the lowest grades, and mixed gender course director teams earning the highest grades (p=0.033).

Review of qualitative comments revealed that there were few explicitly gendered comments made by students in providing faculty feedback. Our team noted that comments about female faculty tended to focus on personality and perceived authority, while comments about male faculty addressed expertise and included more superlatives. For example, students suggested that some female faculty “take more ownership,” appreciated that they were “passionate,” and were “approachable” and “nice.” Male faculty members were more frequently described as “dismissive,” “thoughtful,” “knowledgeable,” and “excellent.”

**Table 2.** Mean faculty grades by gender, degree, rank, and course director team composition within the Clinical Translational Science Track at Mayo Clinic Graduate School of Biomedical Sciences for academic years 2017-2018 and 2018-2019 combined (n=144 faculty*courses, based on 1,352 evaluations).

	Overall (n=144)				p-value (overall)	Female (n=61)				Male (n=83)				p-value (Male/Female)
	n	Mean	Median	SD		n	Mean	Median	SD	n	Mean	Median	SD	
**Overall**	144	3.64	3.72	0.39	-	61	3.72	3.78	0.30	83	3.59	3.67	0.43	0.051*
**Highest Degree**					0.188									0.147
*Masters*	22	3.57	3.59	0.27		2	3.70	3.70	0.05	20	3.56	3.57	0.28	
*MD*	37	3.71	3.80	0.35		7	3.95	4.00	0.13	30	3.65	3.71	0.36	
*PhD*	78	3.65	3.76	0.35		52	3.69	3.78	0.31	26	3.59	3.70	0.41	
*MD/PhD*	7	3.40	3.71	0.93		0	-	-	-	7	3.40	3.71	0.93	
**Rank**					0.969									0.647
*Instructor*	12	3.57	3.57	0.27		0	-	-	-	12	3.59	3.57	0.27	
*Assistant Professor*	23	3.65	3.76	0.34		19	3.71	3.80	0.33	4	3.33	3.31	0.13	
*Associate Professor*	28	3.68	3.78	0.40		18	3.70	3.78	0.32	10	3.64	3.77	0.52	
*Professor*	60	3.73	3.73	0.45		15	3.71	3.78	0.34	45	3.60	3.71	0.48	
*NA*	21	3.71	3.71	0.30		9	3.77	3.74	0.16	12	3.57	3.63	0.35	
**Course Director Team Composition**					0.033*									
*Male (Solo)*	19	3.55	3.58	0.41		-	-	-	-	-	-	-	-	-
*Female (Solo)*	21	3.61	3.61	0.34		-	-	-	-	-	-	-	-	-
*Male (Team)*	42	3.54	3.65	0.46		-	-	-	-	-	-	-	-	-
*Female (Team)*	21	3.82	3.79	0.13		-	-	-	-	-	-	-	-	-
*Mixed Gender Team*	41	3.72	3.88	0.37		-	-	-	-	-	-	-	-	-

## Discussion

Our findings are in stark contrast to those reported previously for faculty in medical schools, in which males receive more favorable grades for their teaching than women ( Morgan *et al.*, 2016). In this study, we found that women in the graduate research program received higher grades on average for teaching than their male counterparts. This is of particular interest as women with advanced degrees in biomedical science are increasingly leaving academia for industry and government due to personal, interpersonal, and professional factors (the proverbial “leaky pipeline”) ( Sarseke, 2018; Ysseldyk *et al.*, 2019). Female faculty in the biomedical research space may face a catch-22 in that, while they are viewed more favorably by their students, they have a steeper climb for promotion and tenure in the academic world than their male counterparts ( Carr *et al.*, 2015). Despite the higher ratings quantitatively, subtle differences in language in student comments reinforce that gender patterns and biases still exist; this has been established previously in several other fields of academia ( Basow *et al.*, 2006).

Furthermore, these results support the fact that teaching teams involving women in the biomedical/translational sciences are rated more highly by students than male or female faculty teaching alone. This is in line with studies that have shown that teams involving women are more successful than those without women, as measured by collective intelligence, and that diversity is associated with creativity and productivity ( Woolley and Malone, 2011; Rock and Grant, 2016; Kubik-Huch *et al.*, 2019). Therefore, seeing this reflected in our education data shows that having women as part of the team and team success translates to graduate biomedical research education.

## Conclusion

This is the first study that our team is aware of to explore gender differences in student evaluations of graduate biomedical research faculty. A strength of this study is that the CCaTS faculty is diverse in their areas of expertise which is reflected in the variety of courses offered through the center; therefore, this study encompasses faculty who teach basic, clinical, population health, and policy-focused courses. Limitations of this study include those associated with small sample sizes, such as limitations in the type of analyses we were able to conduct. Additionally, while the content taught by faculty spans the entire translational spectrum, these findings are only representative of one track within MCGSBS; data were not available school-wide, as CCaTS has led the way with implementing these evaluation procedures. Finally, we did not have data as to which students completed the evaluation surveys, so could not determine whether there were differences on faculty rating based on whether the students were male or female. This factor has been shown in other studies to influence the association between gender and faculty teaching evaluations ( Santhanam and Hicks, 2002; Sprague and Massoni, 2005). Future work should emphasize faculty in more “basic” science settings and include data to explore whether faculty ratings depend on student gender as well as the gender of the faculty, and should be expanded to include non-binary gender categories ( MacNell *et al.*, 2015).

## Take Home Messages

• There may be gender disparities in how students evaluate faculty in graduate biomedical research programs
• This may impact recruitment, retention, and promotion of female faculty
• These disparities may present either as quantitative grading of teaching or more subtly in word choices within qualitative feedback
• Most of these disparities appear to be attributable to implicit biases held by students, rather than explicit biases

## Notes On Contributors

Alexandra Joelle Greenberg-Worisek, PhD, MPH is an Assistant Professor of Epidemiology and the Associate Director for Graduate Curriculum within the Center for Clinical and Translational Science at Mayo Clinic College of Medicine and Science in Rochester, Minnesota, USA.

Katherine Elizabeth Cornelius, MPH, is a program analyst and evaluation specialist within the Center for Clinical and Translational Science at Mayo Clinic College of Medicine and Science in Rochester, Minnesota, USA.

Karen Weavers, MEd, is a program manager within the Office of Applied Scholarship and Education Science at Mayo Clinic College of Medicine and Science in Rochester, Minnesota, USA.

Felicity Enders, PhD, MPH, is a Professor of Biostatistics and the Director for Curriculum within the Center for Clinical and Translational Science at Mayo Clinic College of Medicine and Science in Rochester, Minnesota, USA.

## Declarations

The author has declared that there are no conflicts of interest.

## Ethics Statement

This project was conducted as part of regular educational programming and was deemed by the Mayo Clinic IRB to be a “quality improvement” project.

## External Funding

This publication was supported by Grant Number UL1 TR002377 from the National Center for Advancing Translational Sciences (NCATS). Its contents are solely the responsibility of the authors and do not necessarily represent the official views of the National Institutes of Health.
