# Supplementary material for: Exploring Gender Differences in Student Evaluations of Faculty in a Graduate Clinical Translational Research Program
Source: MedEdPublish (2016). 2020 Mar 11;9:43. [Version 1] doi: 10.15694/mep.2020.000043.1 (PMC10697483; doi:10.15694/mep.2020.000043.1)
Supplement: Peer review history

# Reviewer report: Reviewer response for version 1

**DOI:** 10.21956/mep.19994.r30445

**Published:** 2020-04-03

**Authors:** Shaoting Feng

**Affiliations:** 1 The First Affiliated Hospital of Sun Yat-sen University

This review has been migrated. The reviewer awarded 4 stars out of 5 This study is quite interesting that they examine student evaluations of faculty teaching in a graduate biomedical research training program, using data gathered through Mayo Clinic Center for Clinical and Translational Science (CCaTS) track within the Mayo Clinic Graduate School of Biomedical Sciences (MCGSBS)during two academic years. It indicates that female faculty play such important roles in the teaching team. Looking forward for their further study!

Reviewer Expertise:

NA

No decision status is available

# Reviewer report: Reviewer response for version 1

**DOI:** 10.21956/mep.19994.r30443

**Published:** 2020-03-28

**Authors:** Trudie Roberts

**Affiliations:** 1 Leeds Institute of Medical Education

This review has been migrated. The reviewer awarded 4 stars out of 5 I enjoyed reading this paper and the analysis of the students' comments meant that it is an important contribution to this area. I hope the authors take this work further, possibly by undertaking focus groups with their students to explore in more detail this area of language and why student use the terms they do in relation to gender. Is it merely stereotyping or something more.Using student evaluations for promotional purposes is fraught with issues and concerns about bias but that's another study!

Reviewer Expertise:

NA

No decision status is available

# Reviewer report: Reviewer response for version 1

**DOI:** 10.21956/mep.19994.r30442

**Published:** 2020-03-22

**Authors:** P Ravi Shankar

**Affiliations:** 1 American International Medical University

This review has been migrated. The reviewer awarded 4 stars out of 5 This is an interesting study examining gender differences in student evaluations of faculty. Contrary to earlier published research the authors found female faculty members had higher scores. The authors have mentioned some of the limitations of their study and also mentioned the strengths of the present study. The authors could consider providing some anonymized free text comments of student evaluation of faculty.

Reviewer Expertise:

NA

No decision status is available

# Reviewer report: Reviewer response for version 1

**DOI:** 10.21956/mep.19994.r30440

**Published:** 2020-03-21

**Authors:** Felix Silwimba

**Affiliations:** 1 University of Lusaka

This review has been migrated. The reviewer awarded 5 stars out of 5 this is a good study and is to follow through. it confirms the need for balanced faculty in terms of gender.

Reviewer Expertise:

NA

No decision status is available

# Reviewer report: Reviewer response for version 1

**DOI:** 10.21956/mep.19994.r30439

**Published:** 2020-03-16

**Authors:** Susan Van Schalkwyk

**Affiliations:** 1 Stellenbosch University

This review has been migrated. The reviewer awarded 4 stars out of 5 This article describes a really interesting study and contributes to ongoing work around gender differences and the representation of women in higher education generally. The results are heartening, and the differences between this study and earlier work are interesting. As is indicated in the article, however, further work is now needed to inform our understanding of where these differences come from, and why they are not seen to be filtering through to every day discourse (as represented in the language used in the student comments). However, one obvious difference in this case is that this study was undertaken among postgraduate students versus previous work focusing on undergraduates and I did wonder whether the authors might not have looked to explore that further in the discussion. All in all, provocative early work – I look forward to seeing where it goes from here.

Reviewer Expertise:

NA

No decision status is available

# Reviewer report: Reviewer response for version 1

**DOI:** 10.21956/mep.19994.r30444

**Published:** 2020-03-14

**Authors:** Daniel Xu

**Affiliations:** 1 Curtin Medical School

This review has been migrated. The reviewer awarded 4 stars out of 5 The result of the article echoed the situation in our institution that we have many women as heads of school. Great news that challenges the so-called "glass ceiling" and "leaky pipe".

Reviewer Expertise:

NA

No decision status is available

# Reviewer report: Reviewer response for version 1

**DOI:** 10.21956/mep.19994.r30441

**Published:** 2020-03-13

**Authors:** Irine Sakhelashvili

**Affiliations:** 1 European University

This review has been migrated. The reviewer awarded 5 stars out of 5 Considering the so-called "glass ceiling" as a common barrier in the careers of women around the world, this article is further evidence that the field of biomedical research is also not free from gender stereotypes and bias.Interestingly, the study revealed conflicting results that women at the Mayo Clinic received, on average, higher grades for teaching than their male counterparts; Despite these amazing results, differences in ranks (according to the data presented) confirm the existence of a "glass ceiling", at least as it seems in this school, for example, by the figures, the number of PhDs among women prevails over PhD men, but only one third of women PhD are professors. A gender imbalance in “favor” of women with a lower rank of associate professor is also very noticeable.As you know, an important role in the formation of gender stereotypes and attitudes is played by organizational culture and spirit. There is no need to talk about the necessity to mainstream gender equality at all levels of any structure, of course, this can affect the student's assessment, even at an unconscious level. It would be interesting to continue research on how a student’s gender affects the assessment process.Many thanks to the authors for sharing this very interesting survey. I will wait for further results.

Reviewer Expertise:

NA

No decision status is available
